# Biofluid Markers for Prodromal Parkinson's Disease: Evidence From a Catecholaminergic Perspective

**DOI:** 10.3389/fneur.2020.00595

**Published:** 2020-07-15

**Authors:** Yannick Vermeiren, Yael Hirschberg, Inge Mertens, Peter P. De Deyn

**Affiliations:** ^1^Laboratory of Neurochemistry and Behavior, Department of Biomedical Sciences, Institute Born-Bunge, University of Antwerp, Antwerp, Belgium; ^2^Department of Neurology and Alzheimer Center, University of Groningen and University Medical Center Groningen (UMCG), Groningen, Netherlands; ^3^Centre for Proteomics (CFP), University of Antwerp, Antwerp, Belgium; ^4^Sustainable Health Department, Flemish Institute for Technological Research (VITO), Mol, Belgium; ^5^Department of Neurology, Memory Clinic of Hospital Network Antwerp (ZNA) Middelheim and Hoge Beuken, Antwerp, Belgium

**Keywords:** biomarker, catecholamines, cerebrospinal fluid, DHPG/MHPG, DOPAC, extracellular vesicles, Parkinson's disease, plasma

## Abstract

Parkinson's disease (PD) is the most frequent of all Lewy body diseases, a family of progressive neurodegenerative disorders characterized by intra-neuronal cytoplasmic inclusions of α-synuclein. Its most defining features are bradykinesia, tremor, rigidity and postural instability. By the time PD manifests with motor signs, 70% of dopaminergic midbrain neurons are lost, and the disease is already in the middle or late stage. However, there are various non-motor symptoms occurring up to 20 years before the actual parkinsonism that are closely associated with profound deficiency of myocardial noradrenaline content and peripheral sympathetic denervation, as evidenced by neuroimaging experiments in recent years. Additionally, there is an inherent autotoxicity of catecholamines in the neuronal cells in which they are produced, forming toxic catecholaldehyde intermediates that make α-synuclein prone to aggregation, initiating a cascade of events that ultimately leads to neuronal death. The etiopathogenesis of PD and related synucleinopathies thus may well be a prototypical example of a catecholamine-regulated neurodegeneration, given that the synucleinopathy in PD spreads in synergy with central and peripheral catecholaminergic dysfunction from the earliest phases onward. That is why catecholamines and their metabolites, precursors, or derivatives in cerebrospinal fluid or plasma could be of particular interest as biomarkers for prodromal and *de novo* PD. Because there is great demand for such markers, this mini-review summarizes all catecholamine-related studies to date, in addition to providing profound neurochemical evidence on a systemic and cellular level to further emphasize this hypothesis and with emphasis on extracellular vesicles as a novel diagnostic and therapeutic incentive.

## Introduction

Parkinson's disease (PD) is recognized as the second most common neurodegenerative disorder following Alzheimer's disease (AD), with an approximate incidence rate of 10–18 per 100,000 person-years. PD is present in about 1% of the population over 65 years of age and more than 4–5% in that over 80. Age and gender are established risk factors, followed by ethnicity. An increase by more than 50% is expected by 2030, given the rising life expectancy worldwide (1, 2). Generally, PD is diagnosed when bradykinesia occurs alongside rigidity or tremor, so its clinical diagnosis mostly depends on motor findings. On the pathological level, this is when about 50–80% of the dopaminergic neurons of the substantia nigra pars compacta (SNpc) are lost due to α-synuclein deposits, known as Lewy bodies. PD is, therefore, often diagnosed clinically when the synucleinopathy is already advanced. On the other hand, patients frequently report having non-motor symptoms for 10–20 years before the diagnosis (3). These prodromes, defined as “early (non-specific) symptoms or signs which often indicate the onset of a disease before more diagnostically specific signs and symptoms develop,” provide a potential temporal window during which disease-modifying therapy, once it becomes available, could be administered to prevent or delay the development and progression of disease. Similarly, researchers and clinicians recognize the need for a clinical diagnosis based on quantifiable measures (i.e., biomarkers) to refine qualitative assessments. Characteristic prodromal symptoms of PD are impaired olfaction (anosmia/hyposmia), constipation, depression, excessive daytime sleepiness, rapid eye movement (REM) sleep behavior disorder (RBD), impaired color vision, mild cognitive impairment, and autonomic dysfunction (e.g., orthostatic hypotension (OH), erectile dysfunction, bladder disturbances) (1, 3). From a neurochemical point of view, PD was the first neurodegenerative disease of which the underlying neurochemical abnormality was identified, i.e., striatal depletion of the catecholamine dopamine (DA) (4). This pivotal discovery led to the introduction of the first successful symptomatic treatment with levodopa/carbidopa therapy (5). Almost half a century later, this theory of “central catecholamine deficiency” in PD has expanded considerably, entailing both dopaminergic and noradrenergic neurotransmission deficits, not only in the central but also in the peripheral nervous system.

## Peripheral Catecholaminergic Deficiency as Neurochemical Substrate of Prodromes in PD

A catecholamine is a monoamine neurotransmitter, an organic compound that has a catechol (benzene ring with adjacent hydroxyl groups) and one (“mono”) side-chain amine group. Included among catecholamines are DA and (nor)adrenaline [(N)A]. All are derived from the amino acid tyrosine, which is retrieved from dietary sources, as well as synthesis from phenylalanine. Principal metabolites of DA and (N)A, are 3,4-dihydroxyphenylacetic acid (DOPAC) and homovanillic acid (HVA), and, 3,4-dihydroxyphenylglycol (DHPG) and 3-methoxy-4-hydroxyphenylglycol (MHPG). Furthermore, NA is synthesized from DA (6). Catecholamine neurons are relatively rare in the central nervous system, but with abundant afferent and efferent projections. Toxic intraneuronal α-synuclein depositions in the brainstem nuclei that produce DA and NA, i.e., SNpc and locus coeruleus (LC), therefore, leads to widespread alterations on both central and peripheral levels. Interestingly, upregulated NA reuptake in the LC area of early-stage PD patients, compatible with enhanced NA release, has previously been suggested as a compensatory, protective mechanism against degeneration of nigrostriatal dopaminergic projections (7).

Apart from the central depletion of DA in the brain's nigrostriatal system, giving rise to the well-known motor phenomenology, PD is equally characterized by a severe deficiency of NA in the heart (8). Other Lewy body diseases such as pure autonomic failure or dementia with Lewy bodies (DLB) similarly involve decreased myocardial NA content (9). According to Braak's staging concept, nigrostriatal neuropathological lesioning in PD occurs in stage three out of six (10) and imputes early autonomic involvement (11). The LC, the brain's main source of NA, becomes affected by the synucleinopathy in stage two. During stage one, the dorsal motor nucleus of the vagal nerve becomes affected, which has strong connections with the parasympathetic nervous system of the gastrointestinal system and lungs and with the nucleus ambiguous, which partially involves the innervation of the heart via preganglionic parasympathetic neurons (12). Intriguingly, peripheral cardiac sympathetic neurodegeneration occurs even earlier in the disease process and has a significant clinical importance. The cardiac noradrenergic sympathetic deficiency has been associated with cognitive impairment (13), fatigue/exercise intolerance (14, 15), anosmia (16), RBD (17), visual hallucinations (18), falls from neurogenic OH (19), and decreased survival (20). Gastrointestinal symptoms due to local α-synuclein accumulation and as part of enteric neuronal dysfunction also appear well before the onset of motor symptoms (21).

In PD, OH is a common prodrome that occurs some years before or concurrent with the clinical motor phase and is associated with sympathetic neurocirculatory failure (22). The loss of sympathetic noradrenergic neurons thus might be assumed, but studies using immunoreactive tyrosine hydroxylase as a marker of myocardial catecholaminergic innervation have noted a 75% decrease (23), whereas the loss of NA levels is about 95–99% (8, 9). This points to a proportion of inactive/dysfunctional but not completely eradicated residual nerves and may involve abnormalities in vesicular storage of NA, altered enzymes, decreased vesicular uptake via the vesicular monoamine transporter (VMAT) type 2, and, increased vesicular permeability (24). The cardiac sympathetic denervation does not affect cardiac structure or function under resting conditions but creates a failure to increase the myocardial contractility following stimuli that depend on NA release, for instance during exercise, and is linked with generalized fatigue (25). In AD, myocardial sympathetic innervation is unaltered, so cardiac sympathetic neuroimaging can provide a means for improvement of the differential diagnosis between AD and DLB, as was evidenced previously using ^123^I-metaiodobenzylguanidine scintigraphy (26).

Remarkably, neither the severity of noradrenergic sympathetic denervation nor values for measures of other non-motor manifestations seem to be 100% related to the severity of loss of central nigrostriatal dopaminergic neurons (16, 27). If compatible with Braak's concept of ascending pathology, such patients with decreased striatal dopaminergic innervation should also have cardiac noradrenergic denervation. In contrast, autonomic dysfunction seems to occur independently of the dopaminergic cell loss that causes the parkinsonian triad of motor symptoms (9).

## Low and High Functional Threshold Systems

The hypothesis of Braak et al. of the ascending and trans-synaptically prion-like spreading of α-synuclein from peripheral nerves, such as via a nasal or gastric route (28), to the brainstem and midbrain up to higher cortical structures is questioned due to contradictory neurochemical, neuroimaging, and clinical evidence [(9), for review: (29)]. In part, the evidence against the idea of the trans-synaptic spread comes from Tysnes et al. (30), who concluded that the idea of performing full truncal vagotomy in reducing the risk of PD (31) may be too premature. Recently, Engelender and Isacson (29) challenged the theory of Braak et al. and proposed a model based on evidence of parallel degeneration and pathology of the central and peripheral nervous system in PD. Under this alternative, systems reach their individual thresholds for symptoms at different rates. Even though the brainstem, peripheral and autonomic neurons seem more resilient to insults as opposed to dopaminergic midbrain neurons, the threshold for the brainstem and peripheral motor symptoms to become apparent is lower than that for motor symptoms. The key factor in understanding this theory is the greater functional reserve of the dopaminergic midbrain neurons, which are more sensitive but have vast interconnections throughout the midbrain, striatal, pallidal, thalamic, and cortical nuclei, providing extensive compensatory mechanisms and redundancy to allow initiation of movement. This is in contrast to catecholaminergic neurons of the autonomic, peripheral, and enteric nervous systems which interconnect with the brainstem nuclei (29). Various types of inputs to the striatal medium spiny neurons derived from, e.g., cortex (glutamatergic), thalamus (glutamatergic), and even the dorsal raphe nuclei (serotonergic), could compensate for a progressively reduced input of dopaminergic SNpc neurons. Direct and indirect pathways of the striatal system have strong bidirectional interactions. On the other hand, the functional network of the enteric nervous system (cholinergic, noradrenergic) is much less developed, suggesting that enteric neurons would have less functional reserve and thus may elicit constipation as a prodromal symptom. Cardiac autonomic dysfunction (noradrenergic) and RBD due to lesioning of the brainstem–reticular activating system (cholinergic, noradrenergic) may develop likewise.

**Figure 1:**
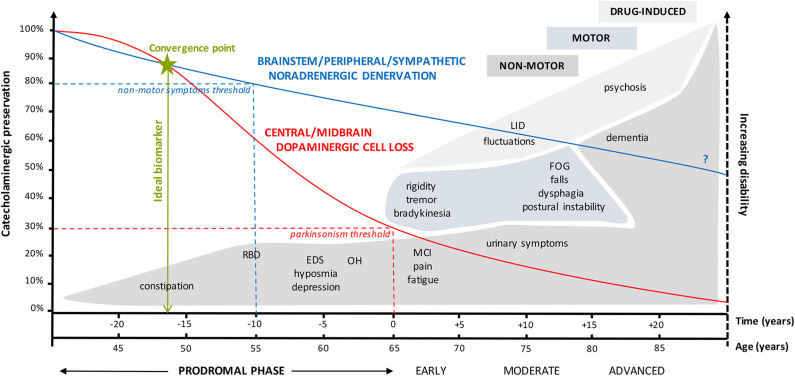
Graphical representation of the complex interplay between catecholaminergic neuronal preservation, the high and low functional threshold theory, and the time course of (non-)motor symptoms in prodromal, early, moderate, and advanced Parkinson's disease. The high threshold for clinical appearance of parkinsonian motor symptoms is only reached when there is at least 70% reduction in dopaminergic midbrain neurons (32). On the other hand, a 20 to 30% loss of mainly noradrenergic brainstem-RAS/peripheral/autonomic/enteric neurons of the low threshold system is already sufficient to elicit a variety of non-motor symptoms such as constipation, RBD, EDS/fatigue, hyposmia, and neurogenic OH. These can be regarded as prodromes. Both catecholaminergic systems progressively degenerate over a period of almost 50 years, albeit with an initial less steeper decline in the noradrenergic system (29). The question mark relates to the uncertainty regarding the extent of noradrenergic decline from early to advanced PD. Figures vary between 40 and 90%, depending on whether NA loss was measured in the myocardium (e.g., 90–95%) or sympathetic ganglia (e.g., 40%). These results are consistent with the concept of centripetal, retrograde “die-back” degeneration of cardiac sympathetic nerves in Lewy body diseases (9). Preferentially, the ideal biofluid marker or combination of markers should be sensitive enough to detect the first subtle catecholaminergic alterations at the convergence point from where both neurotransmitter systems tend to deteriorate in an accelerated fashion. EDS, excessive daytime sleepiness; FOG, freezing of gait; LID, levodopa-induced dyskinesia; MCI, mild cognitive impairment; OH, orthostatic hypotension; PD, Parkinson's disease; RAS, reticular activating system; RBD, REM sleep behavior disorder.

In essence, 70% loss of sensitive dopaminergic midbrain neurons causes motor symptoms (high threshold), whereas only a 20–30% reduction in mainly noradrenergic neurons of the brainstem/peripheral/enteric/autonomic nervous system (low threshold) already seems sufficient to elicit non-motor symptoms. In agreement, the difference between the low and high functional threshold system may explain the appearance of prodromes up to 20 years before the onset of the motor symptomatology and supports the notion of an ideal biofluid marker for prodromal PD to potentially be of catecholaminergic origin (Figure 1).

## The Catecholamine Autotoxicity Theory: Implications for Biomarker Research

Dopamine spontaneously auto-oxidizes to form neuromelanin, the black pigment that pinpoints the SNpc, and the final product of the DA oxidative pathway. Nigral depigmentation, therefore, likely has a neurochemical basis. In a nutshell, the “catecholaldehyde hypothesis” in PD theorizes that long-term increased buildup of 3,4-dihydroxyphenylacetaldehyde (DOPAL)—the catecholaldehyde of DA—significantly contributes to the death of dopaminergic neurons (33, 34). This concept builds on the notion that there is an inherent cytotoxicity of catecholamines and metabolites in the cells in which they are produced [Figure 11 of Goldstein and Sharabi (9)]. The vesicular uptake of cytosolic DA is regulated via VMAT2. Next, DA leaks from the synaptic vesicle into the cytosol, undergoing exocytotic release. DA transporter takes most of the released DA back into the cytosol. However, within the neuron, monoamine oxidase (MAO)-A enzymatically oxidizes DA into an intermittent form, i.e., DOPAL. This reaction yields hydrogen peroxide, which reacts with metal cations to produce extremely harmful hydroxyl radicals. DOPAL can also auto-oxidize, forming DOPAL-quinone, which can be transformed into cysteinyl-DOPAL. Simultaneously, this reaction produces deleterious reactive oxygen species (ROS). Under physiological conditions, DOPAL is metabolized into DOPAC by aldehyde dehydrogenase (ALDH). In glial cells, DOPAC is further metabolized into HVA by catechol-o-methyltransferase (COMT).

Apart from the enzymatic deamination of cytoplasmic DA into DOPAL, DA itself can also auto-oxidize into DA-o-quinone, producing cysteinyl-DA (cys-DA), (amino)chrome, 5,6-indolequinone, polydopamine, and condensation products (e.g., salsolinol), most of which are toxic for the cellular environment. Finally, from 5,6-indolequinone, neuromelanin is formed and stored.

It seems that enzymatic or spontaneous DA oxidation creates aldehydes (DOPAL), ROS, hydrogen peroxide, and thio-catecholamines (cys-DA/cys-DOPAL) within the neuron. The peculiar tendency of α-synuclein to precipitate in dopaminergic neurons can be explained by the fact that DOPAL-quinone can induce oligomerization of α-synuclein in the cytoplasm, forming Lewy bodies. Moreover, ROS inhibit ALDH and thus build up cytoplasmic DOPAL, which leads to an imbalanced system.

The same goes for the production of the noradrenergic aldehyde dihydroxyphenylglycolaldehyde (DOPEGAL). DOPEGAL is formed in the sympathoneural cytosol upon oxidative deamination of NA and undergoes metabolization to form DHPG, mainly via aldehyde/aldose reductase (6). Similar as with DOPAL-induced synucleinopathy in the SN in PD, the DOPEGAL-promoted formation of tau aggregates in the LC in AD has recently been demonstrated (35).

Various early alterations within the dopaminergic neuron, such as decreased vesicular sequestration of DA via VMAT2 and decreased DOPAL metabolism by reduced activity of ALDH, are the multifactorial result of genetic predispositions, exposure to environmental toxins, stress, and aging. Ultimately, this cascade of events could trigger PD pathophysiology. The fungicide benomyl, for instance, increases PD risk by inhibiting ALDH, causing subsequent DOPAL accumulation (36). Furthermore, neuromelanin has the potential to bind environmental redox-active metal ions *in situ*. These are released upon neuronal death, augmenting DOPAL-induced oligomerization of α-synuclein (37, 38).

Altogether, the autotoxicity theory clarifies the selective vulnerability of central and peripheral catecholaminergic neurons, converting a stabile negative feedback-regulated in-cell system to a fragile, unstoppable positive feedback loop (33). This context naturally provides rationale for the development of biofluid markers of dopaminergic/noradrenergic origin.

## Biofluid Catecholamine Markers for Prodromal PD: The Evidence

So far, only a handful of researchers investigated the biomarker potential of circulating catecholamines or derivatives in prodromal or *de novo* PD (dnPD), evidently with no record of present or past therapy with anti-parkinsonian drugs (Table 1). For matters of comparison, neurochemical investigations in early-stage PD patients have also been enlisted in the table.

**Table 1:** Enlistment of studies evaluating biofluid catecholamines and their derivatives or precursors as potential markers for prodromal, *de novo*, or early-stage Parkinson's disease.

**Study**	**Subjects (N)**	**CA marker**	**Main findings**	**Remarks**
Kienzl et al. (39)	15 HC 16 dnPD 21 PD 11 OTHER	Urinary DA-3-o-sulfate and DA-4-o-sulfate	DA-4-o-sulfate is reduced in dnPD compared to HC and OTHER	DA-4-o-sulfate levels remained unaltered during levodopa therapy
D'Andrea et al. (40)	28 HC 16 dnPD 47 nfPD 21 fPD	Plasma octopamine and NA	Octopamine levels were lower in dn/nf/fPD vs. HC; NA levels were only lower in nf/fPD vs. HC	Trace amines are hard to detect; very low circulating levels
Goldstein et al. (41)	38 HC 34 PD (14 dn) 54 MSA 20 PAF	Plasma and CSF l-DOPA, DA, DOPAC, NE and DHPG	CSF DOPAC and DHPG strongly decreased in all groups vs. HC; CSF DOPAC lower, and CSF DHPG higher, in PD than PAF; CSF DOPAC 100% sensitive and 89% specific in dnPD vs. HC	Plasma DHPG levels were lower in PAF than in HC; in PD, CSF and plasma DHPG were positively correlated
Goldstein et al. (42)	26 HC 12 PD+OH 11 PD-OH 21 MSA-p 5 MSA-c 11 PAF	Plasma F-DOPAC and DHPG	F-DOPAC levels were higher, and, DHPG levels lower, in PD+OH vs. MSA-p and HC	F-DOPAC:DHPG ratio differentiated PD+OH from MSA-p
Goldstein et al. (43)	32 HC 24 PD 32 MSA-p 18 PAF	CSF (cys-)l-DOPA, (cys-) DA, DOPAC, (N)A, DHPG	DOPAC was decreased in PD and MSA-p vs. HC	cys-DA:DOPAC two times as high in PD and MSA-p than HC or PAF
Figura et al. (44)	22 early PD 28 aPD+LID 23 aPD-LID	Serum phenylalanine and tyrosine	Phenylalanine levels were higher in early PD than aPD+LID	No differences in tyrosine; no inclusion of HC
Goldstein et al. (45)	26 subjects at risk for PD with 3.7 years FU	CSF l-DOPA and DOPAC	4 out of 26 with low baseline l-DOPA and DOPAC developed PD	At least three risk factors: genetic, olfactory, RBD, and/or OH
Kim et al. (46)	26 untreated dnPD with ±2.5 years of disease duration	Tear fluid DA, NA and A levels	NA and DA were increased, A decreased, in PD vs. HC; increases were pronounced on the ipsilateral motor side	Results were confirmed in (pre)clinical stages of a neurotoxic PD mouse model
D'Andrea et al. (47)	10 HC 21 dnPD 27 PD-treat	Plasma tyrosine, tyramine, tryptamine, octopamine, TRP, β-PEA, 5-HT, NA, MNE	Tyramine differed between all three groups; tyramine, tyrosine and NA combined acted as biomarkers of disease progression	Trace amines are hard to detect; very low circulating levels

*β-PEA, beta phenylethylamine; 5-HT, serotonin (5-hydroxytryptamine); A, adrenaline; aPD+/-LID, advanced PD patients with(out) levodopa-induced dyskinesia; CA, catecholamine; CSF, cerebrospinal fluid; cys-DA, cysteinyl-dopamine; cys-l-DOPA, cysteinyl-levodopa; DA, dopamine; DHPG, 3,4-dihydroxyphenylglycol; dnPD, de novo PD subjects; DOPAC, 3,4-dihydroxyphenylacetic acid; F-DOPAC, fluoro-DOPAC; fPD, fluctuating PD patients; FU, follow-up; HC, healthy controls; l-DOPA, levodopa (3,4-dihydroxyphenylalanine); MNE, metanephrine; MSA, multiple system atrophy; MSA-c, cerebellar variant of MSA; MSA-p, parkinsonian variant of MSA; NA, noradrenaline; nfPD, non-fluctuating PD patients; OH, orthostatic hypotension; OTHER, other neurological disorders, such as polyneuropathy, multiple sclerosis, cerebral infarction, myasthenia gravis, arthritis, cerebral atrophy, and Wilson's disease; PAF, pure autonomic failure; PD, Parkinson's disease; PD-treat, PD patients on levodopa or other dopaminergic drugs; RBD, REM sleep behavior disorder; TRP, tryptophan*.

Kienzl et al. (39) included 16 dnPD patients with Hoehn and Yahr stage scores I or II. The study of D'Andrea and colleagues included 16 dnPD patients diagnosed within 1 year or less following the onset of parkinsonism (40). In 2019, the same group analyzed a larger variety of catecholamines and trace amines, with inclusion of 21 dnPD patients with a disease duration of less than 2 years (47). As for Goldstein et al. (41), 14 out of 34 PD patients were dnPD, with cerebrospinal fluid (CSF) obtained within 2 years or even before the onset of parkinsonism. The authors also excluded DA data from 17 PD patients to eliminate potential treatment effects, even after levodopa washout. Next, the same group performed similar research in early-stage PD patients, complying with three out of four clinical PD criteria, that were off levodopa or MAO inhibitor treatments, as confirmed by additionally measured CSF DOPA levels (42, 43). Figura et al. examined serum amino acids in four dnPD and 18 early PD subjects. The latter group was defined as having a score of I or II on the Hoehn and Yahr staging scale, less than 3 years of disease duration, and stable levodopa response (44). Goldstein et al. (45) also clinically evaluated 26 prodromal subjects who had at least three risk factors for PD, i.e., olfactory dysfunction, RBD, OH, and/or genetic predisposition, and, no motor symptoms. Finally, Kim et al. (46) assessed tear fluid catecholamines in 26 untreated dnPD patients having Hoehn and Yahr stages I–II.

At first glance, (i) three CSF and plasma metabolites [DOPAC (41, 43, 45), DHPG (41, 42), l-DOPA (41, 43, 45)], (ii) one plasma and three tear fluid catecholamines [N(A) (40, 41, 46), DA (46)], and (iii) two derivative plasma trace amines [tyramine (47) and octopamine (40), whether or not combined with plasma NA (47)] seem promising as markers for prodromal or dnPD (Table 1). Regarding CSF DOPAC, results are quite unequivocal, with very low to low levels across PD stages, from prodromal (45) to *de novo* (41) and early-stage (43). For instance, four out of 26 subjects at risk for PD with low CSF levels of DOPAC (<1.22 pmol/ml) and l-DOPA (<2.63 pmol/ml) at baseline inclusion eventually developed PD (45).

Just like DHPG, MHPG—the final metabolite of NA metabolism—has also been indicated as a potential biofluid marker for Lewy body diseases, albeit in the context of cognitive dysfunction in PD (48), or to differentiate DLB from AD (49). Because MHPG crosses both blood–brain (BBB) and CSF–blood barriers (50), plasma alterations may well be indicative of central noradrenergic dysfunction.

## Extracellular Vesicles as A Novel Diagnostic and Therapeutic Approach

It is still challenging to efficiently treat PD patients, due to a BBB impeding passage of most drugs. The development of various drug delivery systems encapsulating, e.g., DA, is, therefore, desirable (51). The focus herein lies upon extracellular vesicles (EVs), nanoparticles including exosomes (<100 nm), microvesicles (100–1,000 nm), and apoptotic bodies (up to 4,000 nm) (52). Their cargo mostly contains lipids, proteins, mRNA, and microRNA (miRNA). EVs show low immunogenicity and can easily cross the BBB. As such, small-molecule therapeutics like paclitaxel, doxorubicin, and curcumin have been encapsulated into exosomes to treat cancer and inflammatory diseases (53–55).

One great advantage over levodopa of an exosomal DA delivery system to the brain via a peripheral route would be that exosomes can be engineered to strategically target specific neurons or neuronal populations (56). This would prevent the non-targeted levodopa-derived DA exocytosis in serotonergic rather than residual dopaminergic nerve terminals. These serotonergic projections innervate entirely different systems, such as the prefrontal cortex, nucleus accumbens, subthalamic nucleus, and hippocampus (57). Since VMAT2 is contained in serotonergic neurons too for the uptake of *in situ* synthesized DA, increased extracellular DA levels in these extrastriatal regions have been linked to various (non-)motor symptoms, such as psychosis and LID (58).

On the other hand, exosomes may intercellularly transfer and sequester misfolded, pathogenic α-synuclein from neuron to neuron and from neuron to glia, in turn leading to the activation of an inflammatory response, and thus contributing to neuronal dysfunction and overall disease progression (59). However, few studies also reported functional evidence of a neuroprotective functionality via exosomal externalization of α-synuclein, which may be beneficial for the surviving dopaminergic neurons of the SNpc (60, 61).

Despite the potential role of EVs in contributing to the onset or progression of PD, exosomes could represent a valuable drug delivery tool (62). Qu et al. (63) administered DA-loaded blood exosomes to PD mice, which successfully entered the nigrostriatal system, induced nigral dopaminergic neurogenesis, and improved the symptomatic performance compared to exogenous DA treatment (64). More recently, Narbute et al. (65) developed EVs derived from stem cells from the dental pulp of human exfoliated deciduous teeth, which are highly proliferative and capable of differentiating into, e.g., neural cells (66–68). These derived EVs were administered intranasally and improved gait parameters in a PD rat model (65).

Furthermore, EVs contain miRNA-124a as part of their cargo (69), which is a potent regulator of MAO-A expression in dopaminergic neurons (70). Regulating EV-containing miRNA-124a expression levels in the brain might be an inventive therapeutic approach, especially because it avoids adverse effects caused by conventional MAO-A inhibitors. Meanwhile, Gui et al. (71) found 16 downregulated miRNAs in CSF exosomes of PD patients compared to controls. Cao et al. (72) concluded that miRNA-19b was downregulated and miRNA-195 and miRNA-24 upregulated in PD. This raises the possibility that serum exosomal miRNA profiling may be a novel strategy for diagnosing prodromal PD.

## Conclusions and Future Perspectives

By and large, in PD, α-synuclein pathologically spreads in synergy with central and peripheral catecholaminergic dysfunctioning, so the quest for biofluid catecholamine markers seems a rational choice. Above, we provided neurochemical, clinical, and pathophysiolocial evidence. On the systemic level, the threshold theory acknowledges that the earliest symptoms are caused by catecholaminergic deficiency of the peripheral/autonomic nervous system, followed by a central dopaminergic depletion. On the cellular level, the hypothesis of DOPAL- and DOPEGAL-induced synucleinopathy and a consequentially altered metabolic route of DA and NA are in agreement with a handful of studies so far that indicated CSF DOPAC and plasma DHPG/MHPG to be of potential interest as biomarkers for prodromal PD in particular. Moreover, the concept of EVs in CSF/plasma needs further refinement, since these nanoparticles may contain a vast amount of information about disease progression, for instance as encoded by exosomal miRNA profiles.

Preferentially, future studies should include more PD patients in prodromal and *de novo* stages based on strict inclusion criteria [e.g., risk factors (45)] and with prolonged follow-up, since current studies only comprised a mere 10–30 study subjects per group. Baseline and intermittent sampling with preceding levodopa washouts would be necessary if early-stage PD subjects are to be included or if treatment was initiated in the meanwhile. Additionally, optimal cutoff CSF/plasma values of DOPAC, l-DOPA, and DHPG/MHPG need to be determined and independently verified on an international scale. In this regard, “very low, low, normal, or high” levels can be attributed to numerical meanings. Metabolomic approaches, such as liquid chromatography with sensitive electrochemical detection, whether or not coupled to mass spectrometry, are routinely implemented in most research hospitals and are feasible techniques for catecholamine biomarker analyses in daily clinical practice (73). Finally, one should also reckon with important methodological issues that are inherent in monoaminergic research, such as sampling conditions (e.g., CSF rostrocaudal concentration gradient, circadian rhythm (74), and dietary effects) and pre-analytical stability of CSF catecholamine metabolites (75).

## Author Contributions

Conceptualization, design, figure, and table: YV. Drafting of original manuscript: YV and YH. Critical manuscript revision and editing: IM and PD. Approval of final version: YV, YH, IM, and PD. Funding acquisition: YV, IM, and PD. All authors agree to be accountable for all aspects with regard to this work.

## Conflict of Interest

The authors declare that the research was conducted in the absence of any commercial or financial relationships that could be construed as a potential conflict of interest.

## References

[1] Kalia LV Lang AE. Parkinson's disease. Lancet. (2015) 386:896–912. 10.1016/S0140-6736(14)61393-3 25904081

[2] Van Den Eeden SK Tanner CM Bernstein AL Fross RD Leimpeter A Bloch DA. Incidence of Parkinson's disease: variation by age, gender, and race/ethnicity. Am J Epidemiol. (2003) 157:1015–22. 10.1093/aje/kwg068 12777365

[3] Rees RN Noyce AJ Schrag A. The prodromes of Parkinson's disease. Eur J Neurosci. (2019) 49:320–7. 10.1111/ejn.14269 30447019 PMC6492156

[4] Ehringer H Hornykiewicz O. Distribution of noradrenaline and dopamine (3-hydroxytyramine) in the human brain and their behavior in diseases of the extrapyramidal system. Wien Klin Wochenschr. (1960) 38:1236–39. 13726012 10.1007/BF01485901

[5] Cotzias GC Levodopa in the treatment of Parkinsonism. JAMA. (1971) 218:1903–8. 10.1001/jama.1971.03190260019005 5171066

[6] Goldstein DS. Catecholamines 101. Clin Auton Res. (2010) 20:331–52. 10.1007/s10286-010-0065-7 20623313 PMC3046107

[7] Isaias IU Marotta G Pezzoli G Sabri O Schwarz J Crenna P. Enhanced catecholamine transporter binding in the locus coeruleus of patients with early Parkinson disease. BMC Neurol. (2011) 11:88. 10.1186/1471-2377-11-88 21777421 PMC3146819

[8] Goldstein DS Sullivan P Holmes C Miller GW Sharabi Y Kopin IJ. A vesicular sequestration to oxidative deamination shift in myocardial sympathetic nerves in Parkinson's disease. J Neurochem. (2014) 131:219–28. 10.1111/jnc.12766 24848581 PMC4241178

[9] Goldstein DS Sharabi Y. The heart of PD: lewy body diseases as neurocardiologic disorders. Brain Res. (2019) 1702:74–84. 10.1016/j.brainres.2017.09.033 29030055 PMC10712237

[10] Braak H Del Tredici K Rüb U de Vos RA Jansen Steur EN Braak E. Staging of brain pathology related to sporadic Parkinson's disease. Neurobiol Aging. (2003) 24:197–211. 10.1016/S0197-4580(02)00065-9 12498954

[11] Del Tredici K Braak H. Lewy pathology and neurodegeneration in premotor Parkinson's disease. Mov Disord. (2012) 27:597–607. 10.1002/mds.24921 22508278

[12] Machado BH Brody MJ Role of the nucleus ambiguus in the regulation of heart rate and arterial pressure. Hypertension. (1988) 11:602–7. 10.1161/01.HYP.11.6.602 2899058

[13] Kim JS Shim Y-S Song I-K Yoo J-Y Kim H-T Kim Y-I. Cardiac sympathetic denervation and its association with cognitive deficits in Parkinson's disease. Parkinsonism Relat Disord. (2009) 15:706–8. 10.1016/j.parkreldis.2009.01.008 19251463

[14] Nakamura T Hirayama M Yasmashita F Uchida K Hama T Watanabe H. Lowered cardiac sympathetic nerve performance in response to exercise in Parkinson's disease. Mov Disord. (2010) 25:1183–9. 10.1002/mds.23127 20629159

[15] Nakamura T Hirayama M Hara T Hama T Watanabe H Sobue G. Does cardiovascular autonomic dysfunction contribute to fatigue in Parkinson's disease? Mov Disord. (2011) 26:1869–74. 10.1002/mds.23744 21542023

[16] Goldstein DS Holmes C Bentho O Sato T Moak J Sharabi Y. Biomarkers to detect central dopamine deficiency and distinguish Parkinson disease from multiple system atrophy. Parkinsonism Relat Disord. (2008) 14:600–7. 10.1016/j.parkreldis.2008.01.010 18325818 PMC2650101

[17] Kim JS Park HE Oh YS Lee SH Park JW Son BC. Orthostatic hypotension and cardiac sympathetic denervation in Parkinson disease patients with REM sleep behavioral disorder. J Neurol Sci. (2016) 15:59–63. 10.1016/j.jns.2016.01.020 26944118

[18] Oka H Yoshioka M Onouchi K Morita M Mochio S Suzuki M. Impaired cardiovascular autonomic function in Parkinson's disease with visual hallucinations. Mov Disord. (2007) 22:1510–4. 10.1002/mds.21581 17516497

[19] Romagnolo A Zibetti M Merola A Canova D Sarchioto M Montanaro E. Cardiovascular autonomic neuropathy and falls in Parkinson disease: a prospective cohort study. J Neurol. (2019) 266:85–91. 10.1007/s00415-018-9104-4 30382389

[20] Goldstein DS Holmes C Sharabi Y Wu T. Survival in synucleinopathies: a prospective cohort study. Neurology. (2015) 85:1554–61. 10.1212/WNL.0000000000002086 26432848 PMC4642141

[21] Edwards LL Pfeiffer RF Quigley EMM Hofman R Balluff M. Gastrointestinal symptoms in Parkinson's disease. Mov Disord. (1991) 6:151–6. 10.1002/mds.870060211 2057006

[22] Goldstein DS Holmes CS Dendi R Bruce SR Li S-T. Orthostatic hypotension from sympathetic denervation in Parkinson's disease. Neurology. (2002) 58:1247–55. 10.1212/WNL.58.8.1247 11971094

[23] Ghebremedhin E Del Tredici K Langston JW Braak H. Diminished tyrosine hydroxylase immunoreactivity in the cardiac conduction system and myocardium in Parkinson's disease: an anatomical study. Acta Neuropathol. (2009) 118:777–84. 10.1007/s00401-009-0596-y 19802627

[24] Goldstein DS Pekker MJ Eisenhofer G Sharabi Y. Computational modeling reveals multiple abnormalities of myocardial noradrenergic function in lewy body diseases. JCI Insight. (2019) 4:e130441. 10.1172/jci.insight.130441 31335324 PMC6777815

[25] Imrich R Eldadah BA Bentho O Pechnik S Sharabi Y Holmes C. Attenuated pre-ejection period response to tyramine in patients with cardiac sympathetic denervation. Ann N Y Acad Sci. (2008) 1148:486–9. 10.1196/annals.1410.066 19120145 PMC4886860

[26] Yoshita M Taki J Yokoyama K Noguchi-Shinohara M Matsumoto Y Nakajima K. Value of 123I-MIBG radioactivity in the differential diagnosis of DLB from AD. Neurology. (2006) 66:1850–4. 10.1212/01.wnl.0000219640.59984.a7 16801649

[27] Goldstein DS Sewell L Sharabi Y. Autonomic dysfunction in PD: a window to early detection? J Neurol Sci. (2011) 310:118–22. 10.1016/j.jns.2011.04.011 21529844

[28] Visanji NP Brooks PL Hazrati LN Lang AE. The prion hypothesis in Parkinson's disease: braak to the future. Acta Neuropathol Commun. (2013) 1:2. 10.1186/2051-5960-1-2 24252164 PMC3776210

[29] Engelender S Isacson O. The threshold theory for Parkinson's disease. Trends Neurosci. (2017) 40:4–14. 10.1016/j.tins.2016.10.008 27894611

[30] Tysnes O-B Kenborg L Herlofson K Steding-Jessen M Horn A Olsen JH. Does vagotomy reduce the risk of Parkinson's disease? Ann Neurol. (2015) 78:1011–2. 10.1002/ana.24531 26418122

[31] Svensson E Horváth-Puhó E Thomsen RW Djurhuus JC Pedersen L Borghammer P. Vagotomy and subsequent risk of Parkinson's disease. Ann Neurol. (2015) 78:522–29. 10.1002/ana.24448 26031848

[32] Ross GW Petrovitch H Abbott RD Nelson J Markesbery W David D. Parkinsonian signs and substantia nigra neuron density in decendents elders without PD. Ann Neurol. (2004) 56:532–9. 10.1002/ana.20226 15389895

[33] Goldstein DS Kopin IJ Sharabi Y. Catecholamine autotoxicity. Implications for pharmacology and therapeutics of Parkinson disease and related disorders. Pharmacol Ther. (2014) 144:268–82. 10.1016/j.pharmthera.2014.06.006 24945828 PMC4591072

[34] Li SW Lin TS Minteer S Burke WJ. 3,4-Dihydroxyphenylacetaldehyde and hydrogen peroxide generate a hydroxyl radical: possible role in Parkinson's disease pathogenesis. Brain Res Mol Brain Res. (2001) 93:1–7. 10.1016/S0169-328X(01)00120-6 11532332

[35] Kang SS Liu X Ahn EH Xiang J Manfredsson FP Yang X. Norepinephrine metabolite DOPEGAL activates AEP and pathological tau aggregation in locus coeruleus. J Clin Invest. (2020) 130:422–37. 10.1172/JCI130513 31793911 PMC6934194

[36] Fitzmaurice AG Rhodes SL Lulla A Murphy NP Lam HA O'Donnell KC. Aldehyde dehydrogenase inhibition as a pathogenic mechanism in Parkinson disease. Proc Natl Acad Sci USA. (2013) 110:636–41. 10.1073/pnas.1220399110 23267077 PMC3545765

[37] Jinsmaa Y Sullivan P Gross D Cooney A Sharabi Y Goldstein DS. Divalent metal ions enhance DOPAL-induced oligomerization of alpha-synuclein. Neurosci Lett. (2014) 569:27–32. 10.1016/j.neulet.2014.03.016 24670480 PMC4061610

[38] Enochs WS Sarna T Zecca L Riley PA Swartz HM. The roles of neuromelanin, binding of metal ions, and oxidative cytotoxicity in the pathogenesis of Parkinson's disease: a hypothesis. J Neural Transm Park Dis Dement Sect. (1994) 7:83–100. 10.1007/BF02260963 7710667

[39] Kienzl E Eichinger K Sofic E Jellinger K Riederer P Kuhn W. Urinary dopamine sulfate: regulations and significance in neurological disorders. J Neural Transm Suppl. (1990) 32:471–9. 10.1007/978-3-7091-9113-2_64 2089110

[40] D'Andrea G Nordera G Pizzolato G Bolner A Colavito D Flaibani R. Trace amine metabolism in Parkinson's disease: low circulating levels of octopamine in early disease stages. Neurosci Lett. (2010) 469:348–51. 10.1016/j.neulet.2009.12.025 20026245

[41] Goldstein DS Holmes C Sharabi Y. Cerebrospinal fluid biomarkers of central catecholamine deficiency in Parkinson's disease and other synucleinopathies. Brain. (2012) 135:1900–13. 10.1093/brain/aws055 22451506 PMC3359749

[42] Goldstein DS Kopin IJ Sharabi Y Holmes C. Plasma biomarkers of decreased vesicular storage distinguish Parkinson disease with orthostatic hypotension from the Parkinsonian form of multiple system atrophy. Clin Auton Res. (2015) 25:61–7. 10.1007/s10286-015-0268-z 25638582 PMC5248558

[43] Goldstein DS Holmes C Sullivan P Jinsmaa Y Kopin IJ Sharabi Y. Elevated cerebrospinal fluid ratios of cysteinyl-dopamine/3,4-dihydroxyphenylacetic acid in Parkinsonian synucleinopathies. Parkinsonism Relat Disord. (2016) 31:79–86. 10.1016/j.parkreldis.2016.07.009 27474472 PMC5125945

[44] Figura M Kuśmierska K Bucior E Szlufik S Koziorowski D Jamrozik Z. Serum amino acid profile in patients with Parkinson's disease. PLoS ONE. (2018) 13:e0191670. 10.1371/journal.pone.0191670 29377959 PMC5788376

[45] Goldstein DS Holmes C Lopez GJ Wu T Sharabi Y. Cerebrospinal fluid biomarkers of central dopamine deficiency predict Parkinson's disease. Parkinsonism Relat Disord. (2018) 50:108–12. 10.1016/j.parkreldis.2018.02.023 29475591 PMC6319386

[46] Kim AR Nodel MR Pavlenko TA Chesnokova NB Yakhno NN Ugrumov MV. Tear fluid catecholamines as biomarkers of the Parkinson's disease: a clinical and experimental study. Acta Nat. (2019) 11:99–103. 10.32607/20758251-2019-11-4-99-103 31993241 PMC6977954

[47] D'Andrea G Pizzolato G Gucciardi A Stocchero M Giordano G Baraldi E. Different circulating trace amine profiles in *de novo* and treated Parkinson's disease patients. Sci Rep. (2019) 9:6151. 10.1038/s41598-019-42535-w 30992490 PMC6467876

[48] van der Zee S Vermeiren Y Fransen E Van Dam D Aerts T Gerritsen MJ. Monoaminergic markers across the cognitive spectrum of Lewy body disease. J Parkinsons Dis. (2018) 8:71–84. 10.3233/JPD-171228 29480224

[49] Janssens J Vermeiren Y Fransen E Aerts T Van Dam D Engelborghs S. Cerebrospinal fluid and serum MHPG improve Alzheimer's disease versus dementia with lewy bodies differential diagnosis. Alzheimers Dement (Amst). (2018) 10:172–81. 10.1016/j.dadm.2018.01.002 29552632 PMC5852321

[50] Sharma RP Javaid JI Faull K Davis JM Janicak PG. CSF and plasma MHPG, and CSF MHPG index: pretreatment levels in diagnostic groups and response to somatic treatments. Psychiatry Res. (1994) 51:51–60. 10.1016/0165-1781(94)90046-9 7910975

[51] Gunay MS Ozer AY Chalon S. Drug delivery systems for imaging and therapy of Parkinson's disease. Curr Neuropharmacol. (2016) 4:376–91. 10.2174/1570159X14666151230124904 26714584 PMC4876593

[52] Gyorgy B Szabo TG Pasztoi M Pal Z Misjak P Aradi B. Membrane vesicles, current state-of-the-art: emerging role of extracellular vesicles. Cell Mol Life Sci. (2011) 16:2667–88. 10.1007/s00018-011-0689-3 21560073 PMC3142546

[53] Sun D Zhuang X Xiang X Liu Y Zhang S Liu C. A novel nanoparticle drug delivery system: the anti-inflammatory activity of curcumin is enhanced when encapsulated in exosomes. Mol Ther. (2010) 9:1606–14. 10.1038/mt.2010.105 20571541 PMC2956928

[54] Tian Y Li S Song J Ji T Zhu M Anderson GJ. A doxorubicin delivery platform using engineered natural membrane vesicle exosomes for targeted tumor therapy. Biomaterials. (2014) 7:2383–90. 10.1016/j.biomaterials.2013.11.083 24345736

[55] Kim MS Haney MJ Zhao Y Mahajan V Deygen I Klyachko NL. Development of exosome-encapsulated paclitaxel to overcome MDR in cancer cells. Nanomedicine. (2016) 3:655–64. 10.1016/j.nano.2015.10.012 26586551 PMC4809755

[56] Wu X Zheng T Zhang B. Exosomes in Parkinson's disease. Neurosci Bull. (2017) 33:331–8. 10.1007/s12264-016-0092-z 28025780 PMC5567508

[57] De Deurwaerdère P Di Giovanni G. Serotonergic modulation of the activity of mesencephalic dopaminergic systems: therapeutic implications. Prog Neurobiol. (2017) 151:175–236. 10.1016/j.pneurobio.2016.03.004 27013075

[58] Navailles S De Deurwaerdère P. Imbalanced dopaminergic transmission mediated by serotonergic neurons in L-DOPA-induced dyskinesia. Parkinsons Dis. (2012) 2012:323686. 10.1155/2012/323686 22007343 PMC3191743

[59] Russo I Bubacco L Greggio E. Exosomes-associated neurodegeneration and progression of Parkinson's disease. Am J Neurodegener Dis. (2012) 1:217–25. 23383394 PMC3560468

[60] Ramirez A Heimbach A Grundemann J Stiller B Hampshire D Cid LP. Hereditary parkinsonism with dementia is caused by mutations in ATP13A2, encoding a lysosomal type 5 P-type ATPase. Nat Genet. (2006) 38:1184–91. 10.1038/ng1884 16964263

[61] Ramonet D Podhajska A Stafa K Sonnay S Trancikova A Tsika E. PARK9-associated ATP13A2 localizes to intracellular acidic vesicles and regulates cation homeostasis and neuronal integrity. Hum Mol Genet. (2012) 21:1725–43. 10.1093/hmg/ddr606 22186024 PMC3465694

[62] Porro C Panaro MA Lofrumento DD Hasalla E Trotta T. The multiple roles of exosomes in Parkinson's disease: an overview. Immunopharmacol Immunotoxicol. (2019) 4:469–76. 10.1080/08923973.2019.1650371 31405314

[63] Qu M Lin Q Huang L Fu Y Wang L He S. Dopamine-loaded blood exosomes targeted to brain for better treatment of Parkinson's disease. J Control Release. (2018) 287:156–66. 10.1016/j.jconrel.2018.08.035 30165139

[64] Borta A Hoglinger GU. Dopamine and adult neurogenesis. J Neurochem. (2007) 3:587–95. 10.1111/j.1471-4159.2006.04241.x 17101030

[65] Narbute K Pilipenko V Pupure J Dzirkale Z Jonavice U Tunaitis V Intranasal administration of extracellular vesicles derived from human teeth stem cells improves motor symptoms and normalizes tyrosine hydroxylase expression in the substantia nigra and striatum of the 6-hydroxydopamine-treated rats. Stem Cells Transl Med. (2019) 5:490–9. 10.1002/sctm.18-0162 PMC6477008 30706999

[66] Miura M Gronthos S Zhao M Lu B Fisher LW Robey PG SHED: stem cells from human exfoliated deciduous teeth. Proc Natl Acad Sci USA. (2003) 10:5807–12. 10.1073/pnas.0937635100 PMC156282 12716973

[67] Sakai K Yamamoto A Matsubara K Nakamura S Naruse M Yamagata M Human dental pulp-derived stem cells promote locomotor recovery after complete transection of the rat spinal cord by multiple neuro-regenerative mechanisms. J Clin Invest. (2012) 1:80–90. 10.1172/JCI59251 PMC3248299 22133879

[68] Jarmalaviciute A Tunaitis V Strainiene E Aldonyte R Ramanavicius A Venalis A. A new experimental model for neuronal and glial differentiation using stem cells derived from human exfoliated deciduous teeth. J Mol Neurosci. (2013) 51:307–17. 10.1007/s12031-013-0046-0 23797732

[69] Karnati HK Garcia JH Tweedie D Becker RE Kapogiannis D Greig NH. Neuronal enriched extracellular vesicle proteins as biomarkers for traumatic brain injury. J Neurotrauma. (2019) 36:975–87. 10.1089/neu.2018.5898 30039737 PMC6444902

[70] Higuchi Y Soga T Parhar IS. Potential roles of microRNAs in the regulation of monoamine oxidase A in the brain. Front Mol Neurosci. (2018) 11:339. 10.3389/fnmol.2018.00339 30271325 PMC6149293

[71] Gui Y Liu H Zhang L Lv W Hu X. Altered microRNA profiles in cerebrospinal fluid exosome in Parkinson disease and Alzheimer disease. Oncotarget. (2015) 6:37043–53. 10.18632/oncotarget.6158 26497684 PMC4741914

[72] Cao XY Lu JM Zhao ZQ Li MC Lu T An XS. MicroRNA biomarkers of Parkinson's disease in serum exosome-like microvesicles. Neurosci Lett. (2017) 644:94–9. 10.1016/j.neulet.2017.02.045 28223160

[73] Havelund JF Heegaard NHH Færgeman NJK Gramsbergen JB. Biomarker research in Parkinson's disease using metabolite profiling. Metabolites. (2017) 7:e42. 10.3390/metabo7030042 28800113 PMC5618327

[74] Janssens J Atmosoerodjo SD Vermeiren Y Absalom AR den Daas I De Deyn PP. Sampling issues of cerebrospinal fluid and plasma monoamines: investigation of the circadian rhythm and rostrocaudal concentration gradient. Neurochem Int. (2019) 128:154–62. 10.1016/j.neuint.2019.04.015 31034914

[75] Willemse EAJ Vermeiren Y Garcia-Ayllon MS Bridel C De Deyn PP Engelborghs S. Pre-analytical stability of novel cerebrospinal fluid biomarkers. Clin Chim Acta. (2019) 497:204–11. 10.1016/j.cca.2019.07.024 31348908

